# A Yeast-Based Screening Unravels Potential Therapeutic Molecules for Mitochondrial Diseases Associated with Dominant *ANT1* Mutations

**DOI:** 10.3390/ijms22094461

**Published:** 2021-04-24

**Authors:** Giulia di Punzio, Maria Antonietta Di Noia, Agnès Delahodde, Carole Sellem, Claudia Donnini, Luigi Palmieri, Tiziana Lodi, Cristina Dallabona

**Affiliations:** 1 Department of Chemistry, Life Sciences and Environmental Sustainability, University of Parma, Parco Area delle Scienze 11/A, 43124 Parma, Italy; giulia.dipunzio@unipr.it (G.d.P.); claudia.donnini@unipr.it (C.D.); cristina.dallabona@unipr.it (C.D.); 2 Laboratory of Biochemistry and Molecular Biology, Department of Biosciences, Biotechnologies and Biopharmaceutics, University of Bari, 70125 Bari, Italy; maria.dinoia@uniba.it (M.A.D.N.); luigi.palmieri@uniba.it (L.P.); 3 Institute for Integrative Biology of the Cell (I2BC), Université Paris-Saclay, 91198 Gif-sur-Yvette, France; agnes.delahodde@i2bc.paris-saclay.fr (A.D.); carole.sellem@i2bc.paris-saclay.fr (C.S.)

**Keywords:** yeast model, mitochondrial diseases, *ANT1* mutations

## Abstract

Mitochondrial diseases result from inherited or spontaneous mutations in mitochondrial or nuclear DNA, leading to an impairment of the oxidative phosphorylation responsible for the synthesis of ATP. To date, there are no effective pharmacological therapies for these pathologies. We performed a yeast-based screening to search for therapeutic drugs to be used for treating mitochondrial diseases associated with dominant mutations in the nuclear *ANT1* gene, which encodes for the mitochondrial ADP/ATP carrier. Dominant *ANT1* mutations are involved in several degenerative mitochondrial pathologies characterized by the presence of multiple deletions or depletion of mitochondrial DNA in tissues of affected patients. Thanks to the presence in yeast of the *AAC2* gene, orthologue of human *ANT1*, a yeast mutant strain carrying the M114P substitution equivalent to adPEO-associated L98P mutation was created. Five molecules were identified for their ability to suppress the defective respiratory growth phenotype of the haploid *aac2^M114P^*. Furthermore, these molecules rescued the mtDNA mutability in the heteroallelic *AAC2/aac2^M114P^* strain, which mimics the human heterozygous condition of adPEO patients. The drugs were effective in reducing mtDNA instability also in the heteroallelic strain carrying the R96H mutation equivalent to the more severe de novo dominant missense mutation R80H, suggesting a general therapeutic effect on diseases associated with dominant *ANT1* mutations.

## 1. Introduction

Mitochondrial diseases are the result of inherited or spontaneous mutations in mitochondrial or nuclear DNA associated with abnormalities of the mitochondrial oxidative phosphorylation (OXPHOS) [1,2,3,4]. OXPHOS is the main source of ATP, and it is therefore not surprising that defects due to these pathologies are most evident in tissues with high energy demands, for example, skeletal muscles, brain, liver, or heart. Several nuclear encoded proteins are implicated in the biosynthesis and the structural composition of OXPHOS pathway, mitochondrial biogenesis, and dynamics and mitochondrial DNA (mtDNA) replication. This explains why most of the mitochondrial disorders are caused by mutations in nuclear DNA. To date, approximately 300 nuclear genes associated with mitochondrial diseases are identified and sorted in broad categories according to their function [4,5,6].

One of these genes is *SLC25A4*, also known as *ANT1*, encoding one of the four isoforms of the ADP/ATP mitochondrial carrier, predominantly expressed in heart, skeletal muscle, and brain [7]. This carrier, one of the most abundant proteins in mitochondrial inner membrane, imports ADP into the mitochondrial matrix, where it is converted to ATP by ATP synthase, and exports ATP to the cytosol [8]. The ANT1 protein belongs to the large family of mitochondrial carriers [9,10]. It presents a six alpha helices transmembrane domain forming a nucleotide translocation channel that alternately cycles between a cytoplasmic and a matrix state during the ADP/ATP exchange [11,12,13].

Mutations in *ANT1* are involved in several degenerative mitochondrial pathologies. Dominant missense mutations (A90D, L98P, D104G, A114P, and V289M) were found in patients affected by adult-onset autosomal dominant progressive external ophtalmoplegia (adPEO), which is clinically characterized by ptosis and weakness of the extraocular muscles [14,15,16,17,18], while recessive missense mutations (A123D and R236P) were found in subjects affected by mitochondrial myopathy and cardiomyopathy [19,20]. De novo dominant missense mutations R80H and R235G were found associated with a marked loss of mitochondrial DNA copy number in skeletal muscle, leading to severe congenital hypotonia and profound muscle weakness [21], whereas a new case of de novo dominant variant (K33Q) was found in a patient with a previously unreported phenotype of mild childhood-onset myopathy [22]. In all cases of mutations associated with *ANT1* gene, deletions or depletion of mtDNA were found in tissues of affected patients. Therefore, although ANT1 protein is not directly involved in mtDNA metabolism, *ANT1* is included in the class of genes whose mutations compromise the maintenance of mtDNA [2,4,5].

Studies on the pathogenic mechanism of *ANT1* mutations were mostly carried out in the yeast *Saccharomyces cerevisiae*, where three genes encoding the ADP/ATP carrier, namely *AAC1*, *AAC2*, and *AAC3*, are present [23,24]. *AAC2* gene, encoding the major isoform of the translocator [23], is considered the yeast ortholog of human *ANT1*. Due to the dispensability of respiratory metabolism and mtDNA, yeast is an excellent model organism to study mitochondrial functions and biogenesis [25,26] and is largely used for the validation of pathological mutations leading to mitochondrial diseases, including mutations in *ANT1* [18,19,21,27,28].

One emerging challenge of experiments by yeast models is their application for discovering drug targets or therapeutic molecules for potential treatment of patients. A phenotype-based approach to identify drugs with potential therapeutic activity for mitochondrial diseases was developed a few years ago [29]. In this yeast-based assay, known as drug drop test, mutant cells unable to grow on respiratory substrates due to mutations in mitochondrial functions are exposed to compounds from chemical libraries spotted on small sterile filters placed on the agar surface. The appearance of a halo of enhanced growth around the corresponding filter is indicative of an effective drug. Currently, the common notion that mitochondrial disorders have no cure is challenged by the proposition of therapeutic approaches tested in suitable cells and model organisms [30]. This yeast-based screening offers the advantage of collecting new therapeutic indications in short time and was already applied with fruitful results [29,31,32,33,34].

Here, we present a similar experimental approach to find therapeutic molecules active against mitochondrial diseases associated with dominant mutations in *ANT1*. Drugs were screened for their ability to suppress the defective respiratory growth of the haploid *aac2^M114P^* yeast mutant carrying the M114P substitution equivalent to L98P in adPEO patients. Five molecules were identified that were able to rescue the OXPHOS phenotypes of this strain. All the drugs were found active to alleviate the mtDNA mutability in the heteroallelic yeast strain *AAC2/aac2^M114P^*. This strain mimics the human heterozygous condition where only one allele is mutated, suggesting a possible therapeutic effect for the tested molecules on adPEO patients. The molecules were effective in reducing mtDNA instability also in the heteroallelic strain carrying the R96H mutation. This mutation is equivalent to the pathological de novo dominant missense mutation R80H, thus expanding the potential therapeutic usage of these molecules also to other non-adPEO pathologies caused by dominant *ANT1* mutations.

## 2. Results

### 2.1. Identification of Molecules Able to Rescue the Oxidative Growth Defect of the aac2^M114P^ Yeast Mutant

In order to identify potentially therapeutic drugs for mitochondrial disorder due to adPEO inducing dominant *ANT1* mutations, we performed a yeast-based pharmacological screening, taking advantage of the haploid strain WB-12/*aac2^M114P^* [27] carrying the mutation equivalent to the aminoacidic substitution L98P in the ANT1 protein. Oxidative growth of this mutant is severely affected but not completely abolished, thus making it a good model for the discovery of rescuing molecules. We used the drug drop test approach [29] to identify which of the 1018 molecules of the Selleck FDA-approved chemical library was able to restore the growth defect. To set up the best experimental conditions, different oxidable carbon sources (ethanol, glycerol, and lactate) were tested, and different amounts of cells were plated. Active compounds were identified after 72–96 h incubation at 28 °C by the halo of growth around the filters where they were deposited.

**Figure 1:**
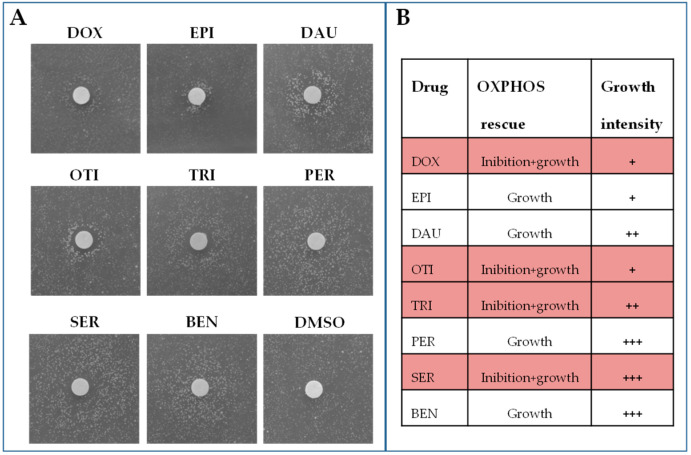
Identification of drugs rescuing the oxidative growth of WB-12/*aac2^M114P^* mutant. (**A**) 4.5 × 10^5^ cells of the WB-12/*aac2^M114P^* mutant strain were spread on a 120 × 120 mm square plate containing solid YP medium supplemented with lactate 2%. Sterile filters were added on the agar surface, and 2.5 μL of the different drugs at the concentration of 10 mM were loaded. One filter was loaded with the same amount of DMSO, the solvent in which the molecules were solubilized, as negative control (C-). Positive hits were identified after 72–96 h incubation at 28 °C by the halo of growth around the filters. (**B**) Rescuing effect on the oxidative growth: +++ Strong effect; ++ Medium effect; + Mild effect. DOX = doxorubicin, EPI = epirubicin HCl, DAU = daunorubicin HCl, OTI = otilonium bromide, PER = pergolide mesylate, TRI = trifluoperazine 2HCl, SER = sertraline HCl, BEN = benzydamine HCl.

To confirm the beneficial effect, the rescuing molecules were tested again (secondary screening) with the same approach but reducing the number of filters present on the plate to avoid any possible interference. The secondary screening led to the identification of eight active compounds: doxorubicin, epirubicin HCl, daunorubicin HCl, otilonium bromide, trifluoperazine 2HCl, pergolide mesylate, sertraline HCl, and benzydamine HCl (Figure 1). Three of them (doxorubicin, epirubicin HCl, and daunorubicin HCl) are chemotherapy medications used to treat cancer and were excluded for further analyses as, due to their high toxicity and side effects, their use is not appropriate for treating mitochondrial diseases that require long-term administration.

Depending on the position of the halo of growth around the filters, active compounds can be classified into two groups:

- Compounds that lead to the formation of a halo of growth near the filter. These molecules have a rescuing effect starting from the maximum concentration tested. This was the case of pergolide mesylate and benzydamine HCl.
- Compounds that lead to the formation of an external crown of growth, where no growth is observed near the filter. These molecules are toxic at high concentrations (near the filter) and active at lower concentrations (far from the filter). This was the case of otilonium bromide, trifluoperazine 2HCl, and sertraline HCl.

### 2.2. Effect of the Identified Drugs on WB-12/aac2^M114P^ Phenotypes

**Figure 2:**
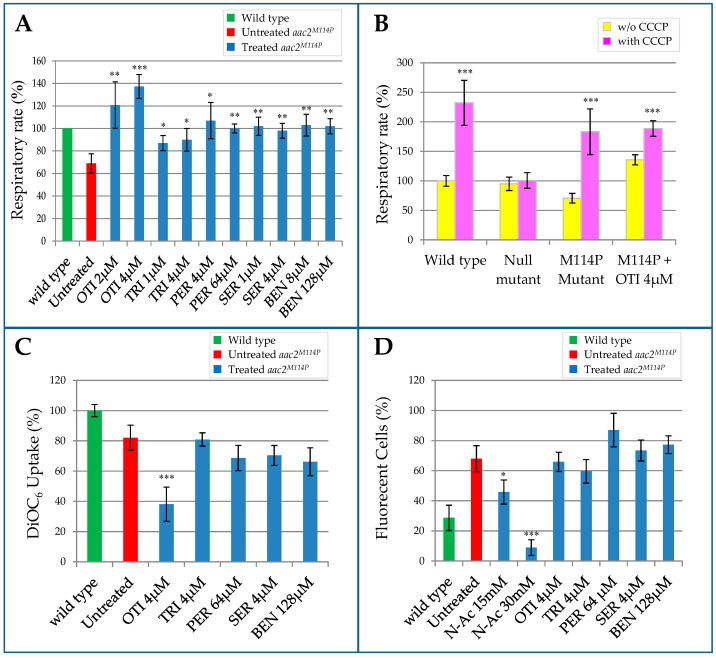
Effect of the identified drugs on WB-12/*aac2^M114P^* phenotypes. Wild type WB-12/*AAC2*, null mutant (*aac2Δ*), and WB-12/*aac2^M114P^* mutant strains with or without the supplementation of active compounds were grown in YP medium supplemented with 0.6% glucose at 28 °C. (**A**) Oxygen consumption rate analysis. The highest and the lowest effective concentrations are reported for each molecule. Values were normalized to the values of the wild type strain and represented as the mean of at least four independent experiments ± SD. (**B**) Oxygen consumption rate under normal (yellow bars) and uncoupled state condition using the mitochondrial uncoupler CCCP (pink bars). Values were normalized to values of the wild type strain without CCCP and represented as the mean of three independent experiments ± SD (**C**) Mitochondrial membrane potential (MMP) was measured by the uptake of the fluorescent dye DiOC_6_. Values were normalized to the values of the wild type strain and represented as the mean of three independent experiments ± SD. (**D**) ROS production was determined as the percentage of fluorescent cells using DHR123 probe. In addition to the shown concentrations, halved concentrations were also tested, and similar results were obtained. The antioxidant agent N-acetyl-cysteine was used at two different concentrations (15 mM and 30 mM) as a positive control. All values are means of three independent experiments ± SD. Statistical analysis was performed using an unpaired, two-tailed Student’s *t*-test, comparing treated (blue bars) versus untreated (red bar) for (**A**,**C**,**D**), and comparing w/o CCCP (yellow bar) versus with CCCP (pink bar): * *p* < 0.05; ** *p* < 0.01; *** *p* < 0.001. The same amount of DMSO (compound vehicle) was added to the untreated mutant (red bar). OTI = otilonium bromide, PER = pergolide mesylate, TRI = trifluoperazine 2HCl, SER = sertraline HCl, BEN = benzydamine HCl.

The WB-12/*aac2^M114P^* mutant strain is characterized by a significative reduction of the respiratory activity [27]. To evaluate the effects of the identified drugs on this phenotype, the strain expressing the *aac2^M114P^* mutant allele was grown for 18 h in the presence or in the absence of the molecules, and oxygen consumption was measured by oxygraph. For every drug, we tested different concentrations below the minimum inhibitory concentration (MIC), i.e., the lowest concentration of the compound that completely inhibits the growth (Supplementary Materials Figure S1). When added, all the molecules led to a significant increase of the respiratory rate, with the level of oxygen consumption being equal to that of the wild type strain. Treatment with otilonium bromide even led to an increase that exceeded the wild type respiratory activity. In Figure 2A, the highest and the lowest effective concentrations for every tested drug are shown. The collected data reflect the results obtained in the drug drop test; some molecules showed a beneficial effect only at a very specific concentration (e.g., otilonium bromide), whereas others were effective on a very broad range of concentrations (e.g., benzydamine HCl) (Figure 2A and Figure S2). To determine whether this increase in oxygen consumption was due to a real beneficial effect of the molecules on respiration activity or to a decoupling effect of the molecule itself, we repeated the experiment by adding the uncoupler carbonyl cyanide 3-chlorophenylhydrazone (CCCP) in the oxygraph chamber. In the presence of CCCP, oxygen consumption was more than doubled both in the wild type and in the WB-12/*aac2^M114P^* mutant (Figure 2B). In contrast, we did not observe any increase in the WB-12 strain carrying the empty plasmid (null mutant). This indicated that the respiration observed in this strain was not coupled to ATP production, thus explaining why the strain was unable to grow on oxidable carbon sources. The addition of the uncoupler led to a significant increase in oxygen consumption for all the tested drugs, with the exception of otilonium bromide (Figure 2B). These data suggested that the mitochondrial membrane of the strain treated with otilonium bromide was partially depolarized.

As in the WB-12/*aac2^M114P^* mutant, where both mitochondrial respiration and ADP/ATP transport were affected, we investigated by flow cytometry the mitochondrial membrane potential (MMP). MMP can be generated by proton pumping in the respiratory complexes or by the electrogenic exchange of ATP^4-^ for ADP^3-^ through the ADP/ATP carrier (in the reverse mode) [35]. For these analyses, we used the lipophilic green-fluorescent dye 3,3P-dihexyloxacarbocyanine iodide (DiOC_6_), whose uptake by mitochondria and consequently its fluorescence emission are modulated by the MMP. The expression on the *aac2^M114P^* mutant allele led to a mild decrease in the mean fluorescence intensity (Figure 2C), indicating that the mutation had a weak negative impact on MMP. We then tested whether this defect could be restored by the addition of the drugs to the culture medium. We did not observe any significative change, except in the case of otilonium bromide that led to a strong reduction of DiOC_6_ uptake. These data are in agreement with the hypothesized depolarization induced by otilonium bromide.

Beside MMP, the production of reactive oxygen species (ROS) is also intimately connected to OXPHOS. In fact, a possible consequence of damage or stalling of the respiratory chain could be an increase in ROS production. In particular, mutations in the Aac2 protein can compromise ADP/ATP translocation activity, leading to a decreased intramitochondrial ADP level. This can, in turn, inhibit the ATP synthase, determining the stalling of the electron flow and an increase in ROS production [19]. To test this hypothesis, we compared ROS production in the mutant WB-12/*aac2^M114P^* and in the WB-12/*AAC2* wild type strain by cytofluorimetric analyses using the fluorescent ROS indicator dihydrorhodamine 123 (DHR123). As shown in Figure 2D, we observed in the mutant strain a doubling of fluorescent cells compared to the wild type, thus demonstrating an increase of ROS production in presence of the *aac2^M114P^* mutant allele. To investigate whether the treatment with the identified molecules could reduce the ROS level, we repeated the cytofluorimetric analyses after incubating the mutant strain with two different concentrations of every drug and, as a positive control, with the well-known antioxidant agent N-acetyl-cysteine. The treatment with N-acetyl-cysteine determined a strong dosage-dependent decrease of ROS, whereas the molecules did not change the percentage of fluorescent cells, indicating that they did not act as antioxidants.

### 2.3. Evaluation of the Drugs Effect on Other aac2 Pathological Mutants

To better characterize the beneficial effects of the five drugs here identified, we tested their effect on other *aac2* mutants.

**Figure 3:**
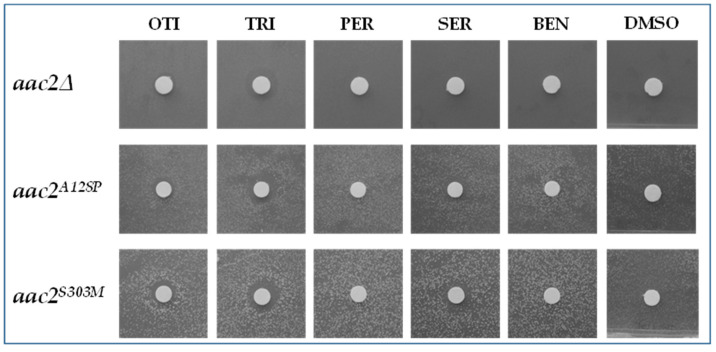
Effect of active compounds on the null *aac2∆* mutant and on the WB-12 strain transformed with *aac2^A128P^* and *aac2^S303M^* mutant alleles. Each filter was spotted with 2.5 µL of the drugs at the following concentrations: OTI = otilonium bromide 2 mM; TRI = trifluoperazine 2HCl 5 mM; PER = pergolide mesylate 25 mM; SER = sertraline HCl 5 mM; BEN = benzydamine HCl 25 mM. DMSO, the compound vehicle, was used as a negative control.

First of all, we tested the effect of the five drugs on the null mutant WB-12. All the drugs were unable to produce a halo of growth around the filter, indicating that they were unable to rescue the oxidative growth phenotype of the null mutant (Figure 3). This result indicated that the drugs exerted their activity when Aac2 protein was present; in other words, drugs were unable to bypass the Aac2 function.

Furthermore, we tested the five drugs on other yeast disease models available in our laboratory, particularly in strains expressing the mutant alleles *aac2^A128P^* and *aac2^S303M^* carrying *aac2* substitutions corresponding to the human pathological mutations A114P and V289M, respectively. These strains were characterized by an oxidative growth that was reduced but not completely abolished [27], similar to the strain WB-12/*aac2^M114P^* used for the library screening. All the drugs, albeit to a different extent, were able to rescue the oxidative defect of the leaky mutants *aac2^A128P^* and *aac2^S303M^* (Figure 3).

We also tested the molecules’ effect on the haploid WB-12 strain carrying the mutant alleles *aac2^R96H^* and *aac2^R252G^*, equivalent to the severe human dominant mutations R80H and R235G, respectively [21]. The oxidative growth of these mutants was completely abolished, as in the null mutant. The drug drop test showed that none of the molecules were able to lead to the formation of a growth halo for either of the two mutants (Figure S3).

As the molecules were able to exert their beneficial effect only on leaky mutants and not on severe mutants or on the null mutant, this indicated that these compounds did not act through a bypass. On the contrary, they were effective only if the Aac2 protein was present and was at least partially functional.

To evaluate if the positive effect of the molecules was due to the increase of Aac2 protein steady-state levels, we performed Western blot analyses on WB-12/*aac2^M114P^* cells grown in the presence or in the absence of the drugs. Aac2 protein quantity was not significantly modified by treatment with any molecule, suggesting that the compounds did not act directly or indirectly by increasing the production of Aac2 protein or by Aac2 stabilization (Figure S4).

The efficiency of ATP and ADP transport of the WB-12/*aac2^M114P^* mutant strain is altered with respect to the wild type [27], therefore, we wondered if the drugs were able to restore, at least partially, this defect. For this purpose, we carried out specific transport assays. The Aac2 protein was solubilized from mitochondria isolated from WB-12 transformants grown in the absence or in the presence of molecules. Proteoliposomes reconstituted with extracts from *aac2^M114P^* mitochondria showed markedly reduced ATP and ADP homoexchanges rates compared with the wild-type transformant, and none of the molecules changed the mutant transport activity (Figure S5). Furthermore, the effect on Aac2 activity was tested by incubating reconstituted proteoliposomes with the drugs prior to transport assays. No significant effect was observed upon pre-incubation with any of the molecules.

### 2.4. Characterization of the Drugs Effect on Caenorhabditis elegans

Yeast being a unicellular organism, we decided to test two beneficial drugs at multi-organ level using the invertebrate *C. elegans*. We selected two drugs with a different effect on growth: otilonium bromide, that determines only a mild oxidative growth improvement in yeast, and pergolide mesylate, that instead shows a strong beneficial effect.

**Figure 4:**
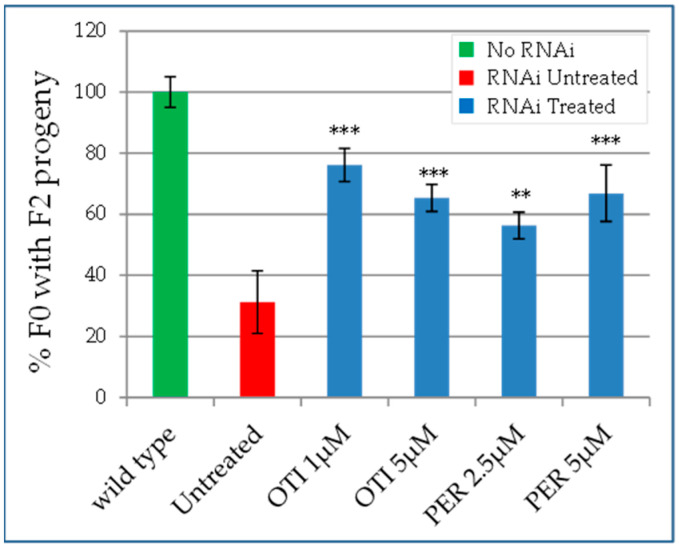
Rescue of embryonic lethality in *C. elegans*. Percentage of F0 adult worms able to give an F2 progeny. The number of F0 adult worms in 8 days treated with RNAi *ant1-1* in the presence of otilonium bromide (OTI) or pergolide mesylate (PER) giving an F2 progeny (blue bars) was compared to that obtained without the addition of the drugs (red bar), where, instead, the same amount of DMSO (compound vehicle) was added. Statistical analysis was performed using Wilcoxon test comparing treated (blue bars) versus untreated mutant (red bar): ** *p* < 0.05; *** *p* < 0.001.

Despite *C. elegans* containing more than one ANT family member, as with most species, including humans, *ant1-1* is the only *ant1* gene that is ubiquitously expressed and strictly required for embryonic and postembryonic development [36]. *ant1-1* depletion was achieved submitting wild type worms from the early stages of development to *ant1-1* RNAi. This led to adults with a reduction in the number of eggs laid and thus in a reduced progeny, as previously described [36,37]. In particular, among the eggs laid by an adult (F0), only 30% developed into adults (F1) able to lay eggs (F2 progeny). In order to determine if otilonium bromide and pergolide mesylate are able to alleviate this phenotype, we followed the progeny of *ant1-1* (RNAi) worms when treated with these drugs. When compared to the development in the absence of drug (vehicle), pergolide mesylate and otilonium bromide significantly increased the percentage of F0 worms able to generate F1 adults laying eggs (F2 progeny) (Figure 4). These data indicated that drug treatment partially rescued the phenotype due to *ant1-1* depletion, in agreement with data obtained in yeast.

### 2.5. Effect of the Identified Drugs on Heteroallelic Strains

The human L98P mutation, associated with adPEO, is dominant. Therefore, the optimal disease model is the so-called heteroallelic strain carrying both a wild type *AAC2* allele and the *aac2^M114P^* mutant allele (*AAC2*/*aac2^M114P^*) [27].

**Figure 5:**
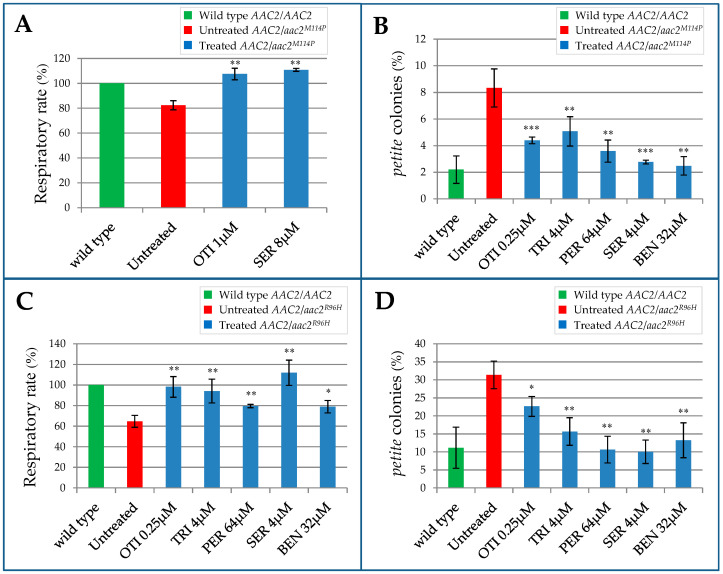
Effect of the identified drugs on the heteroallelic strains. (**A**) Oxygen consumption rate of the *AAC2/aac2^M114P^* strain grown in SC medium (without uracil) at 37 °C supplemented with 0.6% glucose. (**B**) Determination of *petite* frequency of the heteroallelic *AAC2/aac2^M114P^* strain grown in YP medium supplemented with 2% glucose. (**C**) Oxygen consumption rate of *AAC2/aac2^R96H^* strain grown at 37 °C in YP medium supplemented with 0.6% glucose. (**D**) Determination of *petite* frequency of the *AAC2/aac2^R96H^* strain grown in YP medium at 37 °C supplemented with 2% glucose. For the oxygen consumption rate analysis (**A**,**C**), values were normalized to the homoallelic (*AAC2*/*AAC2*) wild type strain and represented as the mean of at least four independent experiments ± SD. For *petite* analysis (**B**,**D**) more than 4000 colonies/strain were scored. All values are means of three independent experiments. Statistical analysis was performed using an unpaired, two-tailed Student’s *t*-test comparing treated (blue bars) versus untreated mutant (red bar): * *p* < 0.05; ** *p* < 0.01; *** *p* < 0.001. OTI = otilonium bromide, PER = pergolide mesylate, TRI = trifluoperazine 2HCl, SER = sertraline HCl, BEN = benzydamine HCl.

The heteroallelic strain *AAC2*/*aac2^M114P^* displays impaired oxygen consumption, indicating that, even in yeast, the mutation behaves as dominant for this phenotype [27]. However, the defect is detectable only at the stress temperature of 37 °C and in stringent nutritional conditions (synthetic medium SC). Analyses performed in the haploid strain WB-12/*aac2^M114P^* showed that, in SC medium, the selected drugs, except otilonium bromide and sertraline HCl, were not effective for the recovery of respiration activity. This different efficiency could be explained by less biodisponibility or by reduced uptake of the drugs in SC medium due to a different composition of the cellular wall. For this reason, in the heteroallelic strain *AAC2*/*aac2^M114P^*, we tested the rescue of the respiratory defect by otilonium bromide and sertraline HCl, and we excluded benzydamine HCl, pergolide mesylate, and trifluoperazine 2HCl. For the analysis, the *AAC2*/*aac2^M114P^* strain was grown in SC in the presence or in the absence of the drugs, and oxygen consumption was then measured by oxygraph. Treatment with both molecules increased significantly the oxygen consumption rate (Figure 5A), indicating that both drugs had a beneficial effect.

Patients affected by adPEO due to *ANT1* mutations are characterized by an alteration of mtDNA maintenance. In yeast, mtDNA instability is associated with the increased segregation of *petite* mutants that arise spontaneously after large deletions or loss of mtDNA, giving rise to respiratory deficiency [38]. Therefore, the effect of a mutation on the structural integrity of the mitochondrial genome can be analyzed by determining the percentage of *petite* colonies produced [39].

As reported in Figure 5B, in the heteroallelic strain *AAC2*/*aac2^M114P^*, the *petite* frequency was markedly increased in comparison with the homoallelic strain *AAC2*/*AAC2*. After determining the optimal working concentration of the molecules in this genetic background, we tested their effect on the reduction of mtDNA instability. All the drugs were able to significantly reduce *petite* percentage of the heteroallelic *AAC2*/*aac2^M114P^* strain (Figure 5B). To exclude the possibility that the beneficial effect observed was due to a selective induction of the *petite* mutant mortality, we performed a *rho^+^/rho^0^* fitness test by growing an equal amount of the wild type W303-1B *rho^+^* and *rho^0^* cells in presence or in absence of the molecule. After 48 h of treatment, the *petite* percentage was not statistically different between treated and untreated culture (Figure S6). This result indicated that the beneficial effect observed on reduction of *petite* percentage should be ascribed to the diminished onset of novel *petite* cells exerted by the molecules, thus confirming a positive effect of the molecules on mtDNA stability.

More recently, the R80H de novo dominant missense mutation associated with a more severe phenotype than adPEO was identified in *ANT1* gene [21]. In order to test if the molecules active on adPEO yeast model could be potentially therapeutic also in the case of this severe mutation, we treated the yeast heteroallelic strain *AAC2*/*aac2^R96H^* available in our laboratory [21,40].

The heteroallelic strain *AAC2*/*aac2^R96H^* displayed a respiratory defect also in YP medium, thus allowing the analysis of all five molecules. The strain was grown for 16 h at 37 °C in the presence or in the absence of the molecules, and oxygen consumption was measured by oxygraph. Addition of all the molecules led to a significant increase of the respiratory rate, bringing the oxygen consumption to a level equal to that of the wild type strain (Figure 5C), thus demonstrating that they were beneficial for this disease model.

Similarly to adPEO patients, patients carrying the R80H mutation are also characterized by mtDNA instability in affected tissue [21], and the same defect is also present in the yeast model [40]. As reported in Figure 5D, *petite* frequency was markedly increased in the heteroallelic strain *AAC2*/*aac2^R96H^* compared to the homoallelic strain *AAC2*/*AAC2*. All the drugs were able to significantly reduce the *petite* percentage of the mutant strain.

## 3. Discussion

The adenine nucleotide translocator (ANT) primary function is to import into the mitochondrial matrix the cytosolic ADP to fuel the ATP production by ATP synthase (complex V) and to export to the cytosol the ATP produced by the OXPHOS. However, the role of ANT is not limited to ADP/ATP transport across the inner mitochondrial membrane (IMM). ANT was shown to contribute to proton loss across the IMM [41] and to the maintenance of the membrane potential in cells lacking mtDNA [42], the regulation of the mitochondrial permeability transition pore [43,44] and the mitochondria-mediated apoptosis [45], and has a role in mitophagy as well [46].

Mutations in *ANT1* gene that codes for one of the four isoforms of the human ADP/ATP carrier are associated with mitochondrial syndromes. Despite the great differences in the clinical manifestation, all patients share the same molecular feature, namely mtDNA defects in affected tissues. The pathogenic mechanism by which mutations in this gene lead to mitochondrial genome deletion/depletion remains to be elucidated.

Notably, no suitable mammal model to study dominant *ANT1* mutations is available. Fibroblasts express the *ANT1* gene at a very low level, while its exogenous expression induces apoptotic cell death [18,47]; mouse myotubes express naturally high levels of *ANT1*, however, no mtDNA deletions or depletion were observed [48]. In contrast, dominant mutations introduced into a wild type yeast *S. cerevisiae*, which mimics human heterozygous condition, recapitulates the patients mtDNA instability, which, in yeast, is associated with the increased segregation of *petite* colonies.

In this report, we exploited a yeast-based screening of drugs, identifying pharmacological compounds able to rescue the defective respiratory growth of a haploid strain carrying the *aac2^M114P^* mutant allele. The drugs that we investigated are currently indicated for treatment of different pathologies: irritable bowel syndrome (otilonium bromide), infections (benzydamine HCl), Parkinson’s disease (pergolide mesylate), schizophrenia (trifluoperazine 2HCl), and depression (sertraline HCl). In human cells, the molecular target of the compounds of repurposing chemical libraries is usually known. The observation that the five selected molecules were able to rescue the OXPHOS phenotypic defect of the *aac2^M114P^* mutant indicated that they are biologically active in yeast. However, yeast does not express the known molecular target of these drugs, thus suggesting the presence of one or more unknown secondary targets.

Since adPEO-inducing mutations are dominant, the best disease model is the heterollelic strain, which carries both a wild type *AAC2* and the *aac2^M114P^* mutant allele and is characterized by mtDNA instability, as found in human patients [27]. All molecules, although with different intensity, were able to increase the respiratory activity and to strongly reduce the mtDNA instability. It should be noted that the selected molecules significantly decreased mtDNA instability without changing mitochondrial ROS content or MMP (except for otilonium bromide), suggesting that ROS overproduction and IMM depolarization cannot be the only mechanisms leading to the onset of *petite* mutants, according to previous observations in *Podospora anserina* [49].

Otilonium bromide was the only compound whose supplementation decreased the MMP, thus indicating a depolarizing activity of this drug. Otiloniun bromide beneficial effect could be due to its ability to reduce the electrochemical gradient across the IMM, protecting the cell from oxidative stress. In fact, mutations in the Aac2 protein compromise ADP/ATP translocation activity, leading to a decreased intramitochondrial ADP level. This can, in turn, inhibit the ATP synthase, thereby blocking the proton flux through the IMM via complex V. Inhibition of the ATP synthase can determine stalling of the electron flow, which, in turn, can lead to increased ROS production [19]. The reason why no reduction of ROS levels was observed after treatment with otilonium bromide remains to be investigated.

In yeast cells, where the known primary target, serotonin/5-HT transporter, is absent, sertraline HCl targets phospholipid membranes of the organelles of the intracellular vesicle transport system [50]. Accordingly, sertraline HCl is placed in the class of drugs CADs (cationic amphiphilic drugs) that interact with phospholipid membranes [51]. The most abundant phospholipid in the IMM is cardiolipin, a critical molecule for formation and stability of respiratory supercomplexes [52,53], including those containing Aac2 protein [54]. In fact, in the yeast *crd1Δ* mutant, devoid of cardiolipin, Aac2 function is compromised [55]. Sertraline HCl could exert its beneficial effect by inducing a variation in mitochondrial membrane cardiolipin content that, in turn, could ameliorate the *aac2* mutant defects by different mechanisms. Recently, it was shown that mutations in the *AAC2* gene cause protein misfolding that might explain the detrimental effect on assembly and stability of multiple protein complexes in the mitochondrial membrane [56]. Accordingly, *aac2* mutant alleles were shown to cause a marked reduction of cytochrome content, suggesting that the Aac2 protein could contribute to the maintenance of the integrity of respiratory complexes in the IMM [27]. It should be underlined that nucleoids, DNA–protein complexes where the mitochondrial genome is packaged, are anchored to the inner membrane [57]. Thus, sertraline HCl, acting on membrane phospholipids composition, could correct the proteostatic stress of the IMM, thus leading to a recovery of both OXPHOS and mtDNA instability defects. Furthermore, since cardiolipin plays a critical role in stabilizing the carrier’s fold state and its transport-related activity [58], a change in its content could partially compensate the altered transport properties of the Aac2 carrier. It should be noted that any change in the lipid milieu of the IMM in vivo would be masked in reconstituted proteliposomes by the large amount of phospholipids, including cardiolipin, that are required to form the artificial vesicles.

Benzydamine HCl, similarly to sertraline HCl, displays membrane-stabilizing properties [59], thus it is reasonable to speculate that it may act by a similar mechanism. Intriguingly, the benzydamine HCl molecular target is not known, but its psychedelic effects at high doses and the structure similarity between this drug and the neurotransmitter serotonin suggest that this compound may share the same target of sertraline HCl, i.e., serotonin/5-HT transporter [60,61].

Trifluoperazine 2HCl and pergolide mesylate are a dopamine receptors antagonist and agonist, respectively. Although trifluoperazine is primarily used as antipsychotic, its antifungal activity makes it useful for treating resistant infections [62,63]. It remains unclear whether its cytotoxicity is mediated by inhibition of calmodulin, a Ca^2+^ binding protein known to be engaged in many regulatory processes, or by an effect on cellular/membrane lipids [64]. Trifluoperazine antifungal action could explain the toxic effects observed on the *aac2* mutant treated with high concentrations of this molecule. Interestingly, treatment of yeast with trifluoperazine was shown to induce a marked increase in intracellular levels of Ca^2+^ [65]. Additionally, for pergolide, an increase in cytosolic calcium was observed both in rat and human muscle cells [66]. Ca^2+^ is fundamental for the adenine nucleotide transport exerted by mitochondrial ATP-Mg/Pi carrier Sal1, whose overexpression is able to rescue the growth defect of cells with impaired ADP/ATP transport activity [67,68,69]. One hypothesis is that trifluoperazine and pergolide may increase Ca^2+^ levels that, in turn, could stimulate Sal1 transport activity, thus overcoming the effects of dominant *aac2* mutations. Alternatively, considering trifluoperazine’s possible effect on membrane lipids, it may act by a similar mechanism proposed for sertraline HCl and benzydamine HCl.

To test the effect of the drugs at a multi-organ level, we exploited the invertebrate *C. elegans* model, where the expression of *ant1-1* was strongly reduced by RNAi. The two tested drugs, otilonium bromide and pergolide mesylate, were able to significantly rescue a worm specific defect, namely embryonic lethality. In this case, the animal model did not express mutations equivalent to those identified in the patients, and for this reason, it cannot be considered a model for the study of *ANT1* dominant mutations. Nevertheless, the results obtained in *C. elegans* can be considered as a proof of concept, indicating that the molecules identified in yeast are active also on a multi-organ animal model and can be potentially applied to higher organisms. Moreover, the observed beneficial effect in the *ant1-1* (RNAi) worm that can be considered similar to a knockout model suggests a potential therapeutic use of these molecules also for cardiomyopathies due to homozygous recessive mutations in *ANT1*.

In conclusion, although the specific molecular mechanism of action of the selected drugs (otilonium bromide, benzydamine HCl, pergolide mesylate, trifluoperazine 2HCl, and sertraline HCl) is not yet identified and requires further investigation, the identification of molecules able to rescue both OXPHOS defect and mtDNA instability could be a starting point for the development of pharmacological therapies for the treatment of adPEO disorder due to *ANT1* mutations. Interestingly, beside adPEO models, all the drugs efficiently reduced mtDNA instability also of the heteroallelic strain carrying the R96H mutation, equivalent to the human R80H mutation, associated with a more severe disease [21], thus expanding the possible applications for the treatment. A limitation of this study is the use of the unicellular yeast model; further studies to test the effect of these molecules on higher cells/organisms are required before clinical trials can be envisaged. It should be noted, however, that our data obtained with the *C. elegans* model, albeit preliminary, are encouraging and usher into further experimentation.

## 4. Materials and Methods

### 4.1. Yeast Strains and Culture Media

Yeast strains used in this study were W303-1B (MAT*a ade2-1 leu2-3,112 ura3-1 his3-22,15 trp1-1 can1-100*) [70] and the isogenic *aac1Δ aac2 Δ* mutant WB-12 (MAT*a ade2-1 trp1-1 ura3-1 can1-100 aac1:: LEU2 aac2:: HIS3*) [71]. WB-12 or W303-1B were transformed with pFL38-*AAC2*, pFL38-*aac2* mutant alleles or the empty vector pFL38 as previously described [21,27].

Cells were cultured in synthetic complete (SC) medium (0.69% YNB without amino acids (Formedium™, Hunstanton, UK) supplemented with 1g/L dropout mix [39] without uracil (to keep the pFL38 plasmid) or in YP (0.5% yeast extract, 1% peptone, Formedium™, Hunstanton, UK). Media were supplemented with various carbon sources at various concentrations as indicated in liquid phase or after solidification with 20 g/L agar (Formedium™, Hunstanton, UK).

### 4.2. High Throughput Screening: Drug Drop Test

The primary screening was performed with the Selleck FDA-approved Drug Library, which contains 1018 compounds. The screening was performed as reported by Couplan et al. (2011) [29]. Specifically, wild-type (WB-12/*AAC2*) and mutant strains (WB-12/*aac2^M114P^*) were grown in YP medium + 2% galactose and incubated at 28 °C in constant shaking. After growth, 4.5 × 10^5^ cells of the WB-12/*aac2^M114P^* mutant strain were spread on 120 × 120 mm square plates containing 90 mL of YP solid medium supplemented with 2% lactate. In total, 31 equidistant sterile 6 mm filters were added on the agar surface and spotted with 2.5 μL of each drug at a concentration of 10 mM dissolved in dimethyl sulfoxide (DMSO), except for one disk, which was soaked with 2.5 μL of DMSO as a negative control (C-). As a positive growth control, wild-type strain (WB-12/*AAC2*) was used, spotting an appropriate number of cells to obtain the same density (cell/cm^2^) of the plated mutant cells. Positive hits were identified after 72–96 h incubation at 28 °C by the halo of growth around the filters.

The secondary screening of positive hits selected by the primary screening was performed in the same conditions, except that four disks were added on each plate, among which was one spotted with DMSO.

### 4.3. Determination of the Minimal Inhibition Concentration

The minimal inhibition concentration (MIC) for each positive molecule was determined by inoculating the wild-type yeast strain WB-12/*AAC2* strain at the concentration of 0.05 OD600/mL in liquid SC or YP medium supplemented with 2% glucose. Molecules were added starting from the maximal concentration at which they were soluble and sequentially halved. Cultures were incubated at 28 °C or 37 °C in constant shaking, and the growth was determined after 24 h by measuring absorbance at 600 nm. MIC was defined as the dosage at which no growth was detectable. As a control, cells were treated with DMSO.

### 4.4. Respiratory Activity

Oxygen consumption rate was measured at 30 °C from yeast cells cultured for 18 h at 28 °C or 16 h at 37 °C in YP or SC medium supplemented with glucose at the non-repressing concentration of 0.6%, until glucose exhaustion using a Clark-type oxygen electrode (Oxygraph System, Hansatech Instruments, King’s Lynn, UK) with 1 mL of air-saturated respiration buffer (0.1 M phthalate–KOH, pH 5.0) and 0.5% glucose. Oxygen consumption was normalized to dry weight, and values were normalized to the wild type strain. CCCP (Sigma-Aldrich^®^, Darmstadt, Germany) was dissolved in DMSO and added to the oxygraph chamber at a final concentration of 10 µM.

### 4.5. Petite Frequency and Fitness Test

For *petite* frequency measurements, cells were pre-grown at 28 °C in SC medium supplemented with 2% ethanol to counter select the *petite* cells already present in the population. Cells were then inoculated at a final concentration of 0.05 OD600/mL in YP liquid medium supplemented with 2% glucose in presence of the molecules or DMSO. After 15 generations, cells were plated on SC agar plates supplemented with 2% ethanol and 0.25% glucose at a dilution giving approximately 200–250 cells/plate. *Petite* frequency was defined as the percentage of colonies showing the *petite* phenotype after 4–5 days incubation at 28 °C [39].

To exclude that the reduction of *petite* colonies observed was due to a negative effect on the division time or on the viability of *petite* mutants, the effect of the drugs on the *petite* fitness was analyzed by growing an equal quantity of *rho^+^* and isogenic *rho^0^* W303-1B cells in the same culture in the presence of the molecules or DMSO. Cells were plated after 15 generations on SC agar plates supplemented with 2% ethanol and 0.25% glucose at a dilution giving approximately 200–250 cells/plate to determine the ratio of respiratory sufficient and respiratory deficient (*petite*) colonies.

### 4.6. Flow Cytometric Analysis

For flow cytometric analysis, cells were pre-grown at 28 °C in SC liquid medium supplemented with 2% glucose and then inoculated at a final concentration of 0.1 OD600/mL in YP supplemented with 0.6% glucose. After 18 h of growth at 28 °C, samples were loaded with the appropriate fluorescent probe. For mitochondrial ROS determination, yeast suspensions were incubated with 1.25 µg/mL of dihydrorhodamine 123 (DHR123; Sigma-Aldrich^®^, Darmstadt, Germany) for 2 h at 28 °C. For mitochondrial membrane potential (MMP) determination, cells were loaded with 0.05 µM of the 3,3P-dihexyloxacarbocyanine iodide (DiOC_6_; Sigma-Aldrich^®^, Darmstadt, Germany) for 30 min at 28 °C. In both cases, at the end of the incubation time, cells were harvested (30 s at 14,000 rpm) and re-suspended in H_2_O, and the fluorescence was quantified by NovoCyte flow cytometer (NovoCyte^®^, ACEA Biosciences, Inc., San Diego, CA, USA). An unstained sample (without DHR123 or DIOC_6_) was prepared as a negative control to set the threshold index, thus delimiting an auto-basal fluorescence area and positive fluorescence. Oxidation of DHR123 by ROS (mainly H_2_O_2_) produced green fluorescent R123 (excitation/emission spectra of 488/530 nm) detected by the fluorescence channel (FL-1) with a 530/30 nm band pass filter. DiOC_6_ fluorescence (excitation/emission spectra of 484/501 nm) was monitored by the same fluorescence channel (FL-1). For each sample, 10,000 cellular events were analyzed. Data achieved from flow cytometer were analyzed using NovoExpress software (NovoExpress^®^, ACEA Biosciences, Inc., San Diego, CA, USA). ROS generation was measured as the percentage of fluorescent cells (PFC) corresponding to cells that produced ROS level increments of at least one log unit [72]. The geometric mean fluorescence intensities of 10,000 cells were used to calculate relative MMP [73]. Values were normalized to the wild type strain.

### 4.7. Protein Extraction and Western Blot Analysis

Total protein of cells grown at 28 °C in YP supplemented with 0.6% glucose were extracted with the trichloroacetic acid (TCA) method by chilling the cells supplemented with 120 mM NaOH, 0.5% β-mercaptoethanol, 650 μM PMSF, and 25% TCA on ice. The proteins were then suspended in Laemmli sample buffer at pH 6.8. Total protein extract from 1.2 OD of cells was loaded on 12% SDS-polyacrylamide gel, and Western blot was performed. Gel was electroblotted onto nitrocellulose filters and sequentially immunostained with specific antibodies against Aac2 and Por1. After incubation with the appropriate HRP-conjugated secondary antibodies, ECL Western blotting substrate (Clarity™, Bio-Rad, Hercules, CA, USA) was used for final detection.

### 4.8. Reconstitution of Aac2 into Liposomes and Transport Measurements

For the isolation of mitochondria, cells were grown at 30 °C in YP medium (1% yeast extract and 2% bactopeptone, pH 4.7) supplemented with 2% galactose as carbon source until an OD of 1.5–1.8 was reached in presence or absence of tested drugs.

Isolation of mitochondria from yeast lysates, solubilization, and incorporation into liposomes of Aac2p were carried out as previously described [27]. For transport measurements, external substrate was removed from proteoliposomes on a Sephadex G-75 column preequilibrated with a buffer containing 50 mM NaCl and 10 mM HEPES, pH 7.5. Transport at 25 °C was started by adding 0.1 mM [^3^H]-ATP (PerkinElmer^®^, Waltham, MA, USA) or [^14^C]-ADP (PerkinElmer^®^, Waltham, MA, USA) to the proteoliposomes and terminated at predetermined time intervals by addition of 30 mM pyridoxal 50 phosphate and 10 mM bathophenanthroline (the “inhibitor-stop” method [74]). The external radioactivity was removed on Sephadex G-75, and the internal radioactivity was measured. In controls, transport was inhibited by the addition of the inhibitors together with the labeled substrate. The transport activity was the difference between experimental and control values. No activity was observed in the absence of added protein or internal substrate.

### 4.9. RNA Interference and Analysis of Embryonic Lethality in C. elegans

The *C. elegans* strain used in this work was the N2 Bristol (wild-type) provided by the Caenorhabditis Genetics Center at the University of Minnesota. L3/L4 worms were obtained from synchronized L1 larvae at 20 °C on NGM agar plates seeded with the *E. coli* strains OP50 following standard protocols [75]. The RNAi experiments were performed using the feeding procedure described by Kamath and Ahringer (2003) [76]. Feeding clone T27E9.1, expressing double-strand RNA for *ant1-1* in HT115 bacteria, was described by Farina et al. (2008) [36] and was verified by DNA sequencing. L4440 control clone contains an empty vector.

RNAi plates (or 12 wells microplates) contained standard NGM plates with the addition of 2 mM isopropyl-β-d-thiogalactopyranoside (IPTG) and 25 μg/mL carbenicillin (final concentrations). According to the conditions, pergolide mesylate or otilonium bromide were added at various concentrations in DMSO. Feeding bacteria (grown for 5 h in LBA liquid medium) were spread and allowed to induce double strand RNA expression for *ant-1* in 24–48 h at 20 °C before L3/L4 wild type larva were transferred. When worms reached adulthood, (F0) adults were picked up and placed individually in fresh RNAi wells of microplates. Each adult was allowed to lay ~20 eggs and then removed. The number of F0 adults having an F2 progeny was determined by counting the number of wells containing at least one F1 adult laying eggs at day 8. In each experiment, we followed 24–48 individual F0 animals per condition, and the experiment was repeated 2–4 times.

## Data Availability

Data sharing not applicable.

## Supplementary Materials

Supplementary materials can be found at https://www.mdpi.com/article/10.3390/ijms22094461/s1.
