# Supplementary material for: A Yeast-Based Screening Unravels Potential Therapeutic Molecules for Mitochondrial Diseases Associated with Dominant ANT1 Mutations
Source: Int J Mol Sci. 2021 Apr 24;22(9):4461. doi: 10.3390/ijms22094461 (PMC8123201; doi:10.3390/ijms22094461)
Supplement: Supplementary file 1 [file ijms-22-04461-s001.zip › ijms-1170506-supplementary.pdf]

SUPPLEMENTARY

	256 µM	128 µM	64 µM	32 µM	16 µM	8 µM	4 µM	2 µM	1 µM	0.5 µM
OTI	-	-	-	-	-	+/-	+	+	+	+
TRI	-	-	-	-	-	+/-	+	+	+	+
PER	+/-	+	+	+	+	+	+	+	+	+
SER	-	-	-	-	-	+	+	+	+	+
BEN	-	+	+	+	+	+	+	+	+	+

No growth (-), reduced growth (+/-), normal growth (+).

Figure S1: Determination of the minimal inhibition concentration (MIC). The wild-type yeast WB-12/*AAC2* strain was inoculated at the concentration of 0.05 OD600/ml in liquid YP medium at 28°C supplemented with 2% glucose. Drugs were added starting from the maximal concentration at which they were soluble and sequentially halved.

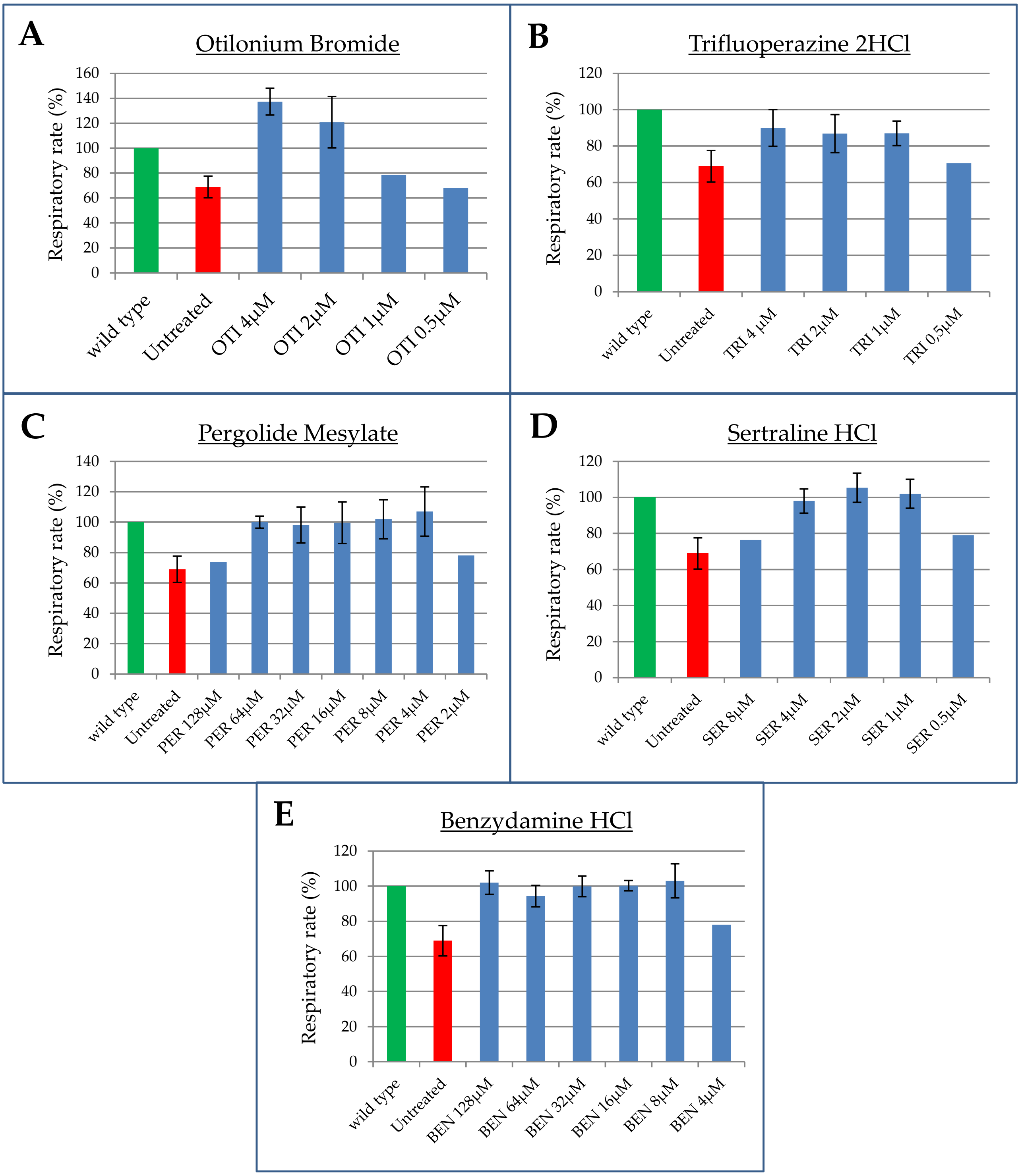

Figure S2: Effect of the identified drugs at different concentrations on WB-12/*aac2*^{M114P} respiratory activity. Wild type WB-12/*AAC2* (green bar) and WB-12/*aac2*^{M114P} mutant strains with (blue bars) or without (red bar) the supplementation of active compounds were grown in YP medium supplemented with 0.6% glucose at 28°C. All values were normalized to the wild type strain and values with error bar are means of at least three independent experiments. The not effective concentrations were not re-tested

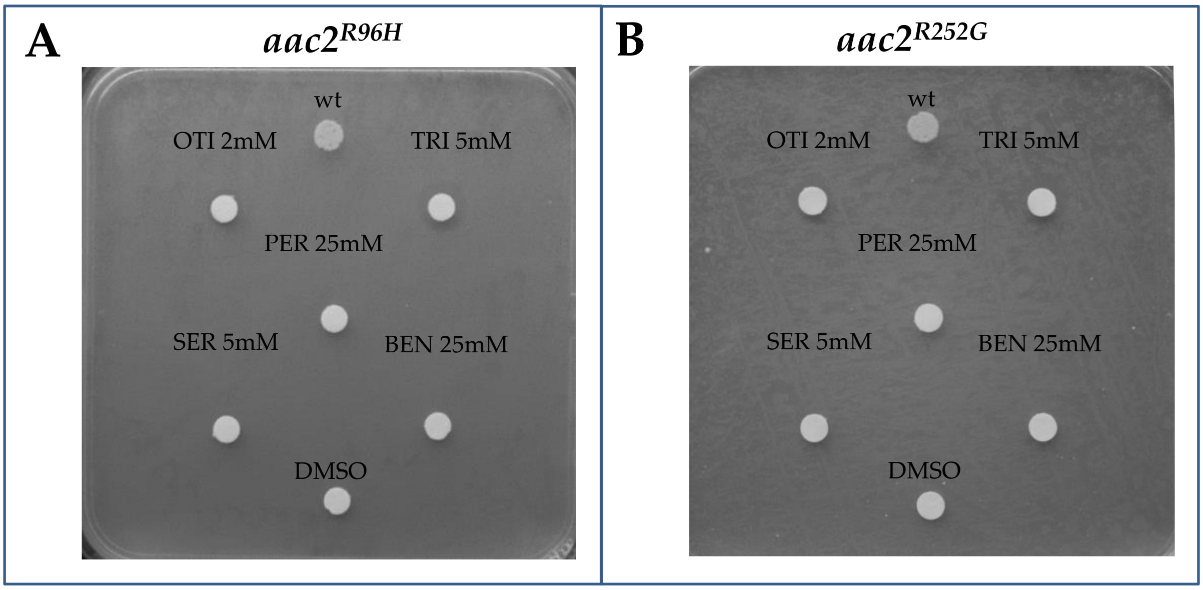

Figure S3: Effect of active compounds on WB-12 strain transformed with *aac2*^{R96H} (A) and *aac2*^{R252G} (B) mutant alleles. Drugs were not effective on both strains. Each filter was spotted with 2.5 µL of the drugs at the indicated concentrations. DMSO, the compound vehicle, was used as a negative control. The wild-type strain WB-12/*AAC2* (wt) was spotted as a positive growth control. OTI= Otilonium Bromide, PER = Pergolide Mesylate, TRI = Trifluoperazine 2HCl, SER = Sertraline HCl, BEN = Benzydamine HCl.

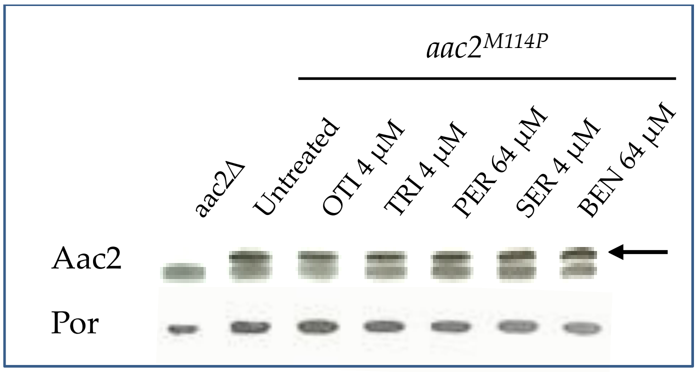

Figure S4: Effect of the identified drugs on Aac2 protein steady-state levels. Western Blot on total protein extracts hybridized by anti-Aac2 antibody. Protein extracts were obtained from WB-12/*aac2*^{M114P} after growth at 28°C for 18h in YP medium supplemented with 0.6% glucose with or without the supplementation of active compounds. The WB-12 strain transformed with the empty vector (*aac2Δ*) was used as negative control. The arrow indicates the signal corresponding to the Aac2 protein, the lower band is an aspecific signal as present also in the null mutants. Porine (Por) was used as a loading control.

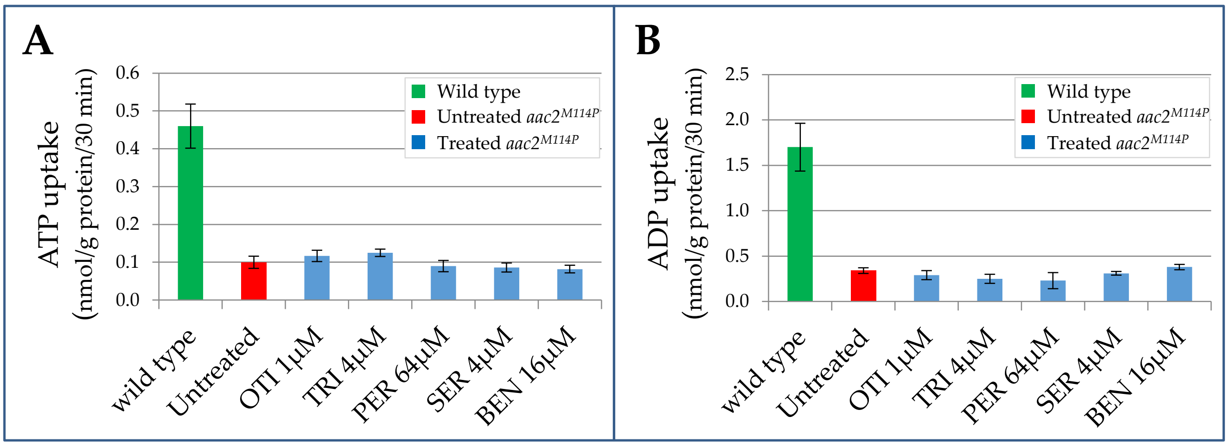

Figure S5: Effect of the identified drugs on the ATP/ADP transport activity. (A) ATP and (B) ADP homo-exchange rates in liposomes reconstituted with extracts of mitochondria isolated from wild type WB-12/*AAC2* (green bar) and WB-12/*aac2*^{M114P} mutant strains grown with (blue bars) or without (red bars) the supplementation of active compounds. To proteoliposomes containing 20 mM ATP or ADP, 0.1 mM [^{3}H] ATP or [^{14}C] ADP respectively was added. The exchange reactions were terminated by adding 30 mM pyridoxal -phosphate and 10 mM bathophenanthroline. Values were represented as the mean of three independent experiments ± SD. Statistical analysis was performed using an unpaired, two-tailed Student'st-test comparing treated (blue bars) versus untreated mutant (red bar). OTI= Otilonium Bromide, PER = Pergolide Mesylate, TRI = Trifluoperazine 2HCl, SER = Sertraline HCl, BEN = Benzydamine HCl.

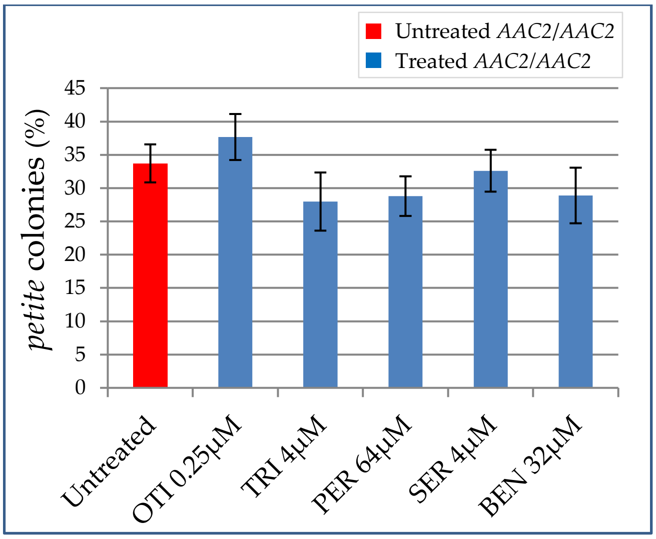

Figure S6: *Petite* frequency obtained by fitness test between *rho*$^+$ e *rho*^{0} yeast strains in presence or in absence of the active compounds. OTI= Otilonium Bromide, PER = Pergolide Mesylate, TRI = Trifluoperazine 2HCl, SER = Sertraline HCl, BEN = Benzydamine HCl.
